# Preparation, Physicochemical Characterization and Oxidative Stability of Omega-3 Fish Oil/α-Tocopherol-co-Loaded Nanostructured Lipidic Carriers

**DOI:** 10.15171/apb.2019.046

**Published:** 2019-08-01

**Authors:** Yaser Shahparast, Masoud Eskandani, Ahmad Rajaei, Ahmad Yari Khosroushahi

**Affiliations:** ^1^Department of Food Science and Technology, Faculty of Agriculture, Shahrood University of Technology, Shahrood, Iran.; ^2^Department of Animal Science, Faculty of Agricultural Sciences, University of Mohaghegh Ardabili, Ardabil, Iran.; ^3^Drug Applied Research Center, Tabriz University of Medical Sciences, Tabriz, Iran.; ^4^Department of Medical Nanotechnology, Faculty of Advanced Medical Science, Tabriz University of Medical Sciences, Tabriz, Iran.

**Keywords:** Co-encapsulation, Omega-3 fish oil, Oxidative stability, Nanostructured Lipid Carriers, α-Tocopherol

## Abstract

***Purpose:*** This study aimed to improve the pharmacokinetic behavior of polyunsaturated fatty acids (PUFAs) oxidation to enhance oxidative stability for inhibiting formation of toxic hydroperoxides, develops off-flavors and shortens shelf-life.

***Methods:*** Nanostructured lipid carrier (NLC) co-encapsulating omega-3 fish oil and α-tocopherol was successfully prepared by melt blending and hot sonication method to enhance the oxidative stability of the fish oil. Encapsulation efficiency (EE) and in vitro release, the oxidative stability of prepared nanoparticles (NPs) were measured using detection of peroxide value (PV) and thiobarbituric acid (TBA) during 40 days.

***Results:*** Electron microscopy and particle size analysis showed dispersed and homogenous NPs with an average diameter of 119 nm. Sustained oil release at a physiologic pH, and longterm stability in terms of the size, zeta, and dispersity of NPs was achieved after 75 days of storage. The omega-3 fish oil co-encapsulated with α-tocopherol in the NLC possessed better oxidative stability compared with the all other formulations. Also, it was found that the NLC as an encapsulation method was more successful to inhibit the formation of the primary oxidation products than the secondary oxidation products.

***Conclusion:*** Generally, these findings indicated that co-encapsulation of fish oil and α-tocopherol within the NLC can be a suitable delivery system in order to enrich foodstuffs, in particular clear beverages.

## Introduction

Nowadays, there are growing needs to fortify foods with bioactive compounds such as omega-3 polyunsaturated oils, oil-soluble vitamins, phytosterols, and carotenoids for developing of novel functional foods that have certain physiological benefits and potential to decrease the risk of some diseases.^1,2^ Beverages are one of the most widely consumed and profitable products in the food industry that can be enriched by nutraceuticals. Many nutraceuticals are non-polar and sparingly water-soluble compounds which cannot simply be dispersed into a polar phase. To this, colloidal delivery systems such as microemulsion, nanoemulsion, or emulsion can be used to overcome the drawback.^3^ omega-3 polyunsaturated fatty acids (PUFAs) are essential in the human diet for optimal brain and cardiovascular health and function. Moreover, findings show that they decrease plasma triglycerides, blood pressure and might also reduce the risk of coronary heart disease, obesity, type 2 diabetes, other inflammatory and autoimmune disorders, and cancer.^4^ Therefore, enrichment of daily foods with PUFAs, especially eicosapentaenoic acid (EPA), docosapentaenoic acid, and docosahexaenoic acid (DHA) which mostly existent in fish oils, is highly desirable. However, successful enrichment of foods with PUFAs always faces different issues, such as their low water solubility and high susceptibility to lipid oxidation under processing and storage conditions that contribute to food deterioration and off-flavors.^5^ Simultaneous use of natural or synthetic antioxidants with PUFAs is a solution for overcoming the mentioned challenges. Among the various antioxidants, vitamin E (α-tocopherol) is an important natural antioxidant in large part due to that it is the main body’s natural antioxidant that can strengthen the immune system and prevent coronary heart disease.^6^ However, antioxidants may create the undesirable color, taste, and flavor in the final product. Among other protective methods, the micro/nano-encapsulation of PUFAs can be considered as a crucial procedure since the method can protect the encapsulated cargo from external oxidative factors such as light, heat, pH, and etc.^2^ In addition, water insolubility of the PUFAs can be resolved by their encapsulation and preparation as emulsions. The nanoemulsions and nanoparticles (NPs) are classified into the particles with a dimension between 1-1000 nm.^7^ As earlier mentioned, beverages can be used for delivery of nutraceuticals to the diet of humans. The appearance of a drink is an effective factor in consumer acceptance. Previous studies showed that reducing the oil droplet sizes to <100 nm produces a translucent emulsion with optical clarity beverages.^8^

Lipid-based nano-carriers have been widely used for encapsulation and delivery of lipophilic and bioactive compounds in food, pharmaceutical and cosmetic industries.^9-12^ Lipid carriers that are commonly used in pharmaceutical and nutraceutical industries can be classified to solid lipid nanoparticles (SLNs) with a solid lipid phase and nanostructured lipid carriers (NLCs) with a combination of solid and liquid lipids.^10,13^

NLCs are composed of a mixture of solid lipid and bioactive liquid oil^14^ that is in a solid state at RT,^15^ and may be of great interest for encapsulation of unstable bioactive compounds. The solid lipid immobilizes the bioactive ingredients and thus enhances its chemical stability through reducing interactions with reactive agents at the interface or surrounding aqueous phase.^16^ Like the fully crystalline SLNs, NLCs possess the physicochemical properties which may directly enhance the bioavailability of bioactive ingredient, enable its sustained release and protect compounds against the light, oxidation, and hydrolysis.^17^ NLCs can be easily engineered based on product requirements via modification in components during preparation.^15^

The oxidation of emulsified oil is proposed to be mechanistically different from bulk oil since the water-oil interface has a pronounced effect on susceptibility to oxidation.^18^ Previous studies have indicated significant differences in the oxidative stability among emulsions, SLNs and NLCs.^17,19^ Recently, NLCs containing PUFAs have been prepared and findings showed significant protective effects for PUFAs against oxidization.^15^ Previous studies showed that bioactivity of a nutraceuticals cargo might be enhanced by co-encapsulation with the antioxidants.^7,20^

This study aimed to optimize for the first time lipophilic omega-3 fish oil, as the main ingredient, and α-tocopherol, as natural antioxidant, loaded NLCs (omega-3 fish oil /α-tocopherol-loaded NLCs) composed of Precirol^®^ATO5 as a lipid matrix and poloxamer 407 as emulsifier to improve the pharmacokinetic behavior of PUFAs and to enhance oxidative stability of omega-3 fish oil.

## Materials and Methods

### Materials

Fish oil (35% PUFA) containing 17% EPA, 11% DHA and 0.12% free fatty acids. The peroxide value (PV) of oil was 1.3 equivalent of peroxides/kg of oil, and anisidine value was 13.9 according to the specifications of the manufacturer) was purchased from Alhavi Company (Iran). The fish oil was stored in a freezer at 75°C until use. Tween 20, tween 60, tween 80, lecithin, sodium dodecyl sulfate (SDS), poloxamer 407, Precirol^®^ATO5 (glyceryl palmitostearate), stearin, palmitic acid, 1,1,3,3-tetraethoxypropane and α-tocopherol were provided from Sigma Aldrich (Steinheim, Germany). Chloroform, methanol, ammonium thiocyanate, Iron (II) and cumene hydroperoxide were purchased from Merck (Germany). All materials were used without further purification. Also, ultra-pure water was used throughout the study.

### Preparation of lipid particles

The type of solid lipid and surfactant for preparation of NLC were optimized and selected based on the preliminary investigation (data not shown). For preparing the NLCs, first solid lipid was fully melted in a water bath at 85°C and heated omega-3 fish oil (4 min, 85°C) was dispersed in solid lipid by stirring and the ratio of solid lipid phase to liquid lipid phase (fish oil) was 7:3. Next, the poloxamer 407 solution as aqueous phase surfactant (85°C), was added to the hot lipid mixture. Lipid and aqueous phases were sonicated at 85°C, for 10 minutes using a Sonics VCX-400 sonicator (Sonics & Materials Inc., Newtown, Conn., USA). Finally, the emulsion was sealed and cooled for lipid solidification and recrystallization in an ambient temperature to form the NLC dispersions. During the investigation, ultrasonic power (100 and 400 W) and the ratio of emulsifier to a solid phase (1:1 and 2:1) effects on the particle size and appearance of samples were investigated. Omega-3 fish oil/α-tocopherol-co-loaded NLCs also were prepared by the same previous method with omega-3 fish oil containing 100 ppm α-tocopherol. In the preparation of all samples, the steps mentioned earlier were fixed.

### Determination of particle size, zeta potential, morphology and stability of NLC

The particle size of lipid NPs like the hydrodynamic diameters (z-average), and polydispersity index (PDI) of NLC dispersions were determined by dynamic light scattering (DLS) (Zetasizer Nano ZS, Malvern Instruments Ltd., Worcestershire, UK), at a scattering angle of 90°C and at a temperature of 25°C (pH 7.0). To prevent multiple scattering effects and achieve an adequate scattering intensity prior to the measurement, dispersed samples were diluted in deionized water and then were subjected to analysis. The particle size analysis using intensity distribution are reported as the mean hydrodynamic diameter (z-average) based on Stokes-Einstein equation and the PDI, which ranges from 0 (monodisperse) to 1.0 (very broad distribution) were calculated based on three individual measurements.

The electrophoretic mobility (zeta potential) of the NLC containing omega-3 fish oil was measured with the same device by using electrophoretic light scattering procedure. Before zeta potential measurements, the samples were diluted 1:100 with sodium phosphate buffer (10 mM, pH 7) and placed in a capillary cell (DTS 1070, Malvern Instruments, Malvern, UK). The zeta potential was calculated based on the Helmholtz–Smoluchowski equation.

$$EE(\%)=(\frac{W_{i}-W_{f}}{W})\times 100$$

Morphology of the NLC containing omega-3 fish oil was achieved using a transmission electron microscopy (JEOL USA Inc., Peabody, MA, USA). The sample was diluted 100 times in ultra-pure water and negatively stained with phosphotungstic acid, and then dried on copper grids at room temperature.

In order to evaluate the physical stability of the NLCs during storage (25 °C in darkness), the particle size of the NLCs was determined after 75 days.

### Entrapment efficiency of fish oil

The omega-3 fish oil entrapment efficiency of the optimized NLC was determined by measuring the amount of the un-encapsulated fish oils by using Amicon ultra centrifugal filter units (Ultra-15, MWCO 10 kDa, Sigma–Aldrich) and was quantified using spectrophotometrically. For this, the optimized NLC was diluted with distilled water (1:4). Then, the sample was kept in the upper section of the ultra-centrifuge tube and centrifuged at 12 000 rpm for 15 min. The collected sample containing the free omega-3 fish oil was measured spectrophotometrically at 475 nm using a double-beam UV-2450 (Shimadzu, USA). The entrapment efficiency (EE) was calculated using the following equation.

where “W_i_” is the mass of initially added fish oil in the formulation and “W_f_” is the mass of unloaded fish oil.

### Assessment of fish oil release rates from nanoparticles

The release kinetics of the fish oils were investigated using dialysis bag method in the phosphate buffer solution pH 7.4 during a 2-week test.^21^ The spectroscopic reading at 475 nm (λ_max_ of fish oil) was performed on the sample at predetermined time intervals and the plot between cumulative amounts of omega-3 fish oil release vs. time was drawn.

### Differential scanning calorimetry

A differential scanning calorimeter (DSC-60, Shimadzu, Kyoto, Japan) was used to determine the melting and crystallization behavior of the samples. 2 mg of the formulations were placed in aluminum pans, and hermetically sealed. As a reference, empty pans were used. The samples were heated from 20 to 300°C with a heating rate of 20°C/min.

### Lipid oxidation products

The fresh NLC containing 100 ppm α–tocopherol (10 mL), NLC without α–tocopherol and fish oil in water emulsion with a ratio of poloxamer 407: oil (1:1) were placed in a sealed screw-cap glass tube and kept in an oven for 40 days at 25°C. A sufficient amount of samples was taken out in a fixed time to measure the primary and secondary oxidant products.

#### Primary lipid oxidation products

The primary oxidation products were determined following the procedure as described by Salminen et al.^17^ To determine the PV, the sample was added to 9.8 mL chloroform: methanol (2.3:1, v/v) and mixed for 5 seconds on a vortex mixer. Then, ammonium thiocyanate solution (50 µL) was added to the mixture. After then, iron (II) solution (50 µL) was added. Iron (II) solution was prepared by mixing BaCl_2_ and FeSO_4_ to final concentrations of 0.132 and 0.144 mol L^−1^, respectively. After 5 min incubation at room temperature, the absorbance of the samples was measured at 500 nm against a blank by a Shimadzu-1800 UV–visible spectrophotometer (Shimadzu, Tokyo, Japan). The quantities of lipid hydroperoxides were calculated using an external standard curve made of cumene hydroperoxide.

#### Secondary lipid oxidation products

The secondary oxidation products were monitored by quantification of the thiobarbituric acid (TBA) reactive substances (TBARS) as described by Wang et al.^22^ In brief, 0.2 mL of the samples were mixed with 1.8 mL of deionized water and 4.0 mL of TBA solution. TBA solution was prepared by dissolving 15 g of trichloroacetic acid (15% w/v) and 0.375 g of TBA (0.375%) in 100 mL of 0.25 mol L^−1^HCl. In the following, the mixtures were heated in a boiling water bath for 15 min and then cooled to room temperature. Finally, the mixtures were centrifuged (2000 g for 15 min). The intensity of the color created as a result of the reaction between TBA with malondialdehyde (MDA), an important by-product of lipid peroxidation, was measured at 532 nm. The standard curve of 1,1,3,3-tetraethoxypropane was used to determine the MDA concentrations.

### Statistical analysis

All measurements were performed in triplicate. Means and standard deviations (SD) were calculated using Microsoft Excel 2010 (Microsoft, Redmond, WA, USA). The lipid oxidation results were analyzed with a one-way ANOVA followed by Duncan post-hoc test using SPSS 16.0 (SPSS Inc., Chicago, IL, USA). Differences at *P* ≤ 0.05 were considered to be significant.

## Results and Discussion

### Preparation of NLC

#### Selection of solid lipid and surfactant

Omega-3 fish oil-loaded NLC was prepared using simple hot ultra-sonication methods. The type of lipid and surfactant used was selected based on an optimization of prepared NPs in terms of particle size less than 100 nm, the stability of NPs and acceptable EE % (data not shown). For this purpose, the effects of different types of solid lipids (stearin, palmitic acid, and Precirol ATO5) and emulsifier (tween 20, tween 60, tween 80, lecithin, SDS and poloxamer 407) on the stability and particle size were investigated. Preliminary analyses showed that Precirol ATO5 (as solid lipid) was best suited for the preparation of stable, dispersed NLCs. The same result was obtained by Liu and Wu.^23^ They optimized the formulation of NLC containing lutein with different solid lipids (stearic acid, myristic acid, palmitic and Precirol ATO5) and found that only Precirol ATO5 was able to produce stable NPs. It should be pointed out that lutein like as omega-3 fish oil is a relatively poorly water-soluble compound and may exhibit a similar physicochemical characteristic. Surfactants have an important role in stabilizing NPs in colloidal systems and prevent particles aggregation during the time. Surfactants also may control the crystallization process of NLCs.^24^ Our preliminary analyses showed that NPs prepared using poloxamer 407 were stable during storage, spherical and their size was less than 100 nm particle. Poloxamer 407 is a hydrophilic non-ionic surfactant with HLB number 22 and block copolymer of polyethylene oxide and polypropylene oxide. The hydrophobic (polypropylene oxide) chains adsorb on the particle surfaces as the anchor chain, while the hydrophilic (polyethylene oxide) chains pull out from the surface to the aqueous medium, creating a stabilizer layer.^25^

#### Effect of ultrasonic power on the particle size distribution

**Figure 1:**
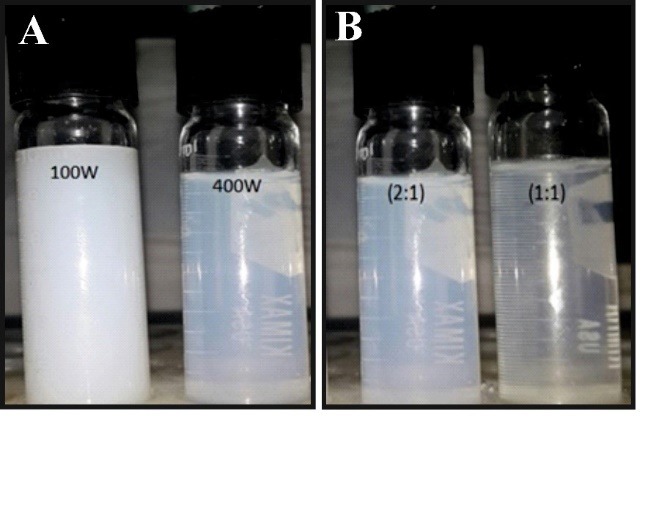
Images showing (A) ultrasonic power effects on the appearance of omega-3 fish oil, and (B) appearance of nanostructured lipid carriers with poloxamer 407 to solid phase ratios of 1:1 and 2:1.

For studying the effect of ultrasonic power on the particle size and dispersity, NLCs containing omega−3 fish oil were prepared at two ultrasonic power of 100 and 400 W. Table 1 show the particle size distribution of the NLCs prepared by different ultrasonic power. As it is clear, the particle size at 400 W ultrasonic power was smaller (202 nm) than those obtained at 100 W (639 nm). In addition, the higher ultrasonic power led to lower PDI and transparent NPs. It should be pointed out that the transparent appearance of NLC is a positive feature that is expected during formulation of beverages for better optical clarity (Figure 1).

**Table 1:** The effects of “ Poloxamer 407 to solid phase ratio” and “Ultrasonication Power” on size and dispersity of omega-3 fish oil-loaded NLCs

**Formulation code**	**Poloxamer 407 to solid phase ratio**	**Ultra sonication Power**	**Size ± STD**	**PDI ±STD**
1	(2:1)	100	639.4 ± 0.95	0.563 ± 0.04
		400	202.2 ± 0.47	0.575 ± 0.03
2	(1:1)	400	119.4 ± 0.12	0.12 ± 0.03

#### Effect of surfactant concentration on the particle size distribution

Two NLC formulations prepared with a different ratio of poloxamer 407: lipid phase (2:1 and 1:1) and fixed ultrasonic power of 400 W were compared in terms of their PDI and NPs size. The equal amount of poloxamer 407 and lipid phase during NPs formulation lead to monodispersed NLCs (0.32 ± 0.03) and smaller NPs (119 nm ± 0.12) when compared to the formulations which the amount of poloxamer 407 was twofold higher than lipid phase (Table 1). In addition, the NLC with less emulsifier was more transparent than the NLC with more emulsifier. This result shows that the amount of emulsifier has an important role in the particle size and appearance of NLC formulation containing omega-3 fish oil. However, more emulsifier leads to polydispersed and large NPs.

#### Zeta potential and morphology of particles in the optimized formulation

**Figure 2:**
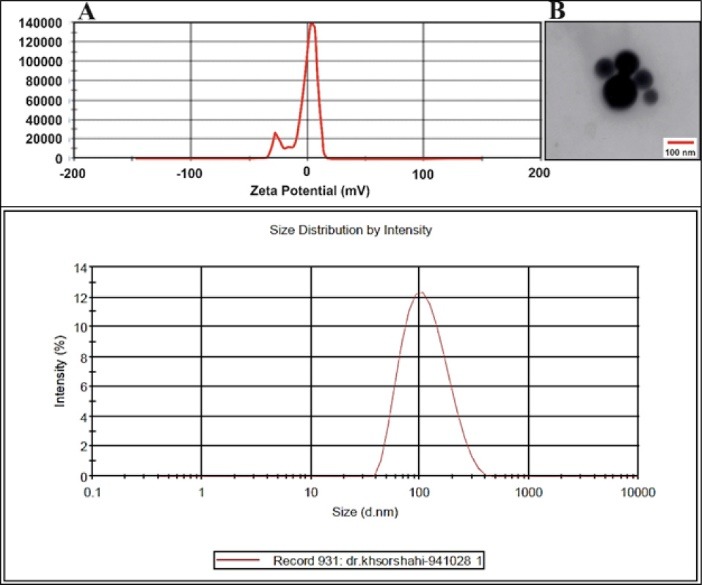
Images showing (a) zeta potential, (b) transmission electron microscopy, and (c) particle size distribution analysis of the optimized nanostructured lipid carrier.

The zeta potential is an important indicator of the stability of colloidal dispersions. Generally, the higher/lower zeta potential (>30 mV and <-30 mV) values cause stable colloidal systems.^26^ The zeta potential of the optimized NLC formulation (Formulation code 2) was measured at water solution at pH 7 using Zetasizer (Malvern Zetasizer, Nano ZS90, Malvern instruments Ltd., UK). Figure 2a shows that the zeta potential of NLCs was very small (close to zero) in large part due to the nonionic nature of poloxamer 407 and other constituents of the system. Regardless, the stability of NLCs could be due to the polymeric and bulky structure of poloxamer 407 as well as the steric repulsion of the surfactant molecules in the systems. Therefore, it seems that the electrostatic repulsion of the NPs has no critical role in the stability of prepared NPs. Polyhydroxy surfactants stabilize systems by creating spatial exclusion and due to their non-ionic nature, low and zero zeta potential would be obtained.^27,28^

The morphology and surface rheology of the prepared NPs was investigated using transmission electron microscopy. TEM image of the optimal NLC is presented in Figure 2b. As it is clear, the NLCs were spherical, and their diameter was 60–200 nm confirming previously obtained results by DLS.

#### Stability of NLC

In order to study the stability of NLC during storage, the optimized formulation was made without and also with 100 ppm α-tocopherol. In the following, the NLC suspensions kept for 75 days at 25ºC and particle size distribution of NLC was measured. Particle size and PDI of optimized NLC obtained at the first and end days storage are shown in Table 2. The results showed that the changes in the mean particle size and PDI were very little after 75 days of storage. Generally, it can be concluded that optimal NLC had a good stability.

**Table 2:** Changes in optimal nanoparticle systems are shown as results comparison after system preparation and 75 days as characterized by particle size, polydispersity index (PDI) and zeta potential

**Formula (NLC)**	**Time (day)**	**Mean size (nm)**	**PDI**	**ζ potential [mV]**
Fish oil	1	101	0.17	-1.13
	75	100	0.15	-1.13
Fish oil+100ppm α-tochopherol	1	119	0.12	-1.05
	75	110	0.13	-1.1

#### Entrapment efficiency and fish oil release rate from nanoparticles

**Figure 3:**
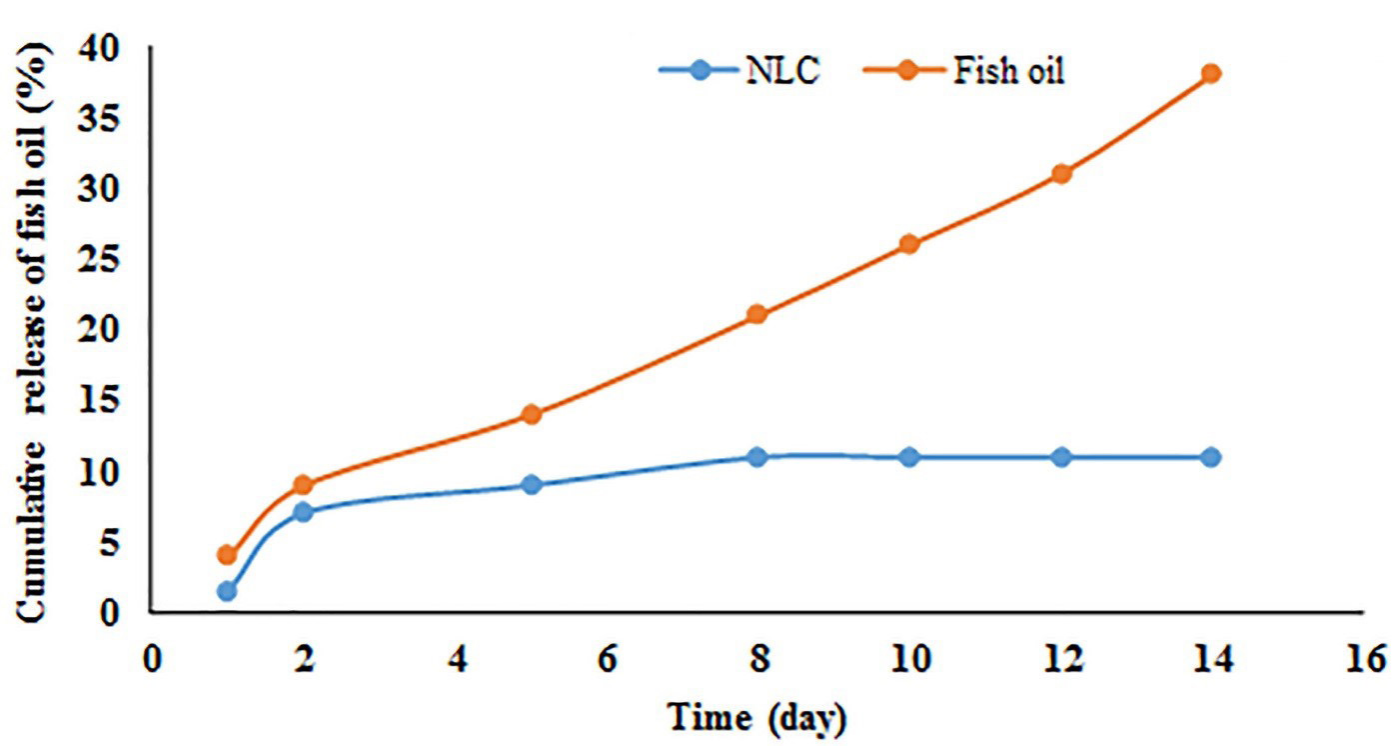
In vitro release of fish oil from nanostructured lipid carrier during storage at ambient temperature.

The entrapment efficiency of optimized NLC containing omega-3 fish oil was 30 ± 0.4% indicating it was able to participate in the structure of NLC as a liquid lipid phase. In the following, the release pattern of omega-3 fish oil from the optimized NLC during storage are shown in Figure 3. It was found that the rate and extent of omega-3 fish oil release became constant after 8 days. No omega-3 fish oil release was observed after 8 days of storage. This result suggests that probably all of the omega-3 fish oil is not well covered with Precirol ATO5 as a solid phase and poloxamer as a surfactant.

#### Thermal behavior of lipid particles

The DSC is the most important method for studying melting and crystallization behavior of lipid particles. Solid lipid NPs based on solid lipids or blend of solid lipids show limited bioactive compound loading and release during storage mainly due to an ongoing crystallization process. While the liquid lipids are incorporated into the solid lipids and hence the particles exhibit less crystalline structure. Also, greater temperature difference leads to the greater lipid crystals disorders. This phenomenon causes a maximum encapsulation efficiency (EE) for NLC system.^29^

**Figure 4:**
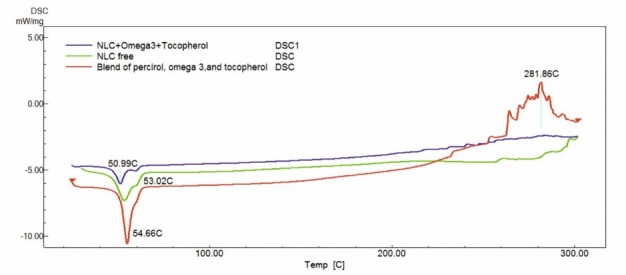
Differential scanning calorimetry thermogram of blank nanostructured lipid carrier (NLC), co-encapsulated NLC and a physical blend of Precirol ATO5, omega-3 fish oil, and α-tocopherol.

Figure 4 shows DSC curves of the physical mixture and lyophilized NPs with or without α-tocopherol. As it is clear, one melting point was observed at 54.66°C for the physical mixture sample. The peak of melting point in the NLC loaded without α-tocopherol showed a shift from 54.66°C to 53.02°C. This result showed that an interaction between omega-3 fish oil and Precirol ATO5 occurred after preparing NLC, resulting in a depression of the melting point. α-tocopherol loaded-NLC showed a melting point peak at 50.99°C. It is clear that the degree of crystallinity of Precirol ATO5 was depressed by omega-3 fish oil and α-tocopherol in the form of NLC. The above results further confirmed that both omega-3 fish oil and α-tocopherol could decrease crystallinity and increased less ordered modification of Precirol ATO5. These results showed that compounds in the optimized NLC had a good compatibility with each other and created a new composition with new thermal properties.

#### Oxidative stability of lipid particles

The objective of these experiments was to compare the oxidative stability of omega-3 fish oil in forms of a different formulation including NLC and emulsion, and also to determine the impact of α-tocopherol on lipid oxidation in the NLC incorporated with omega-3 fish oil.

**Figure 5:**
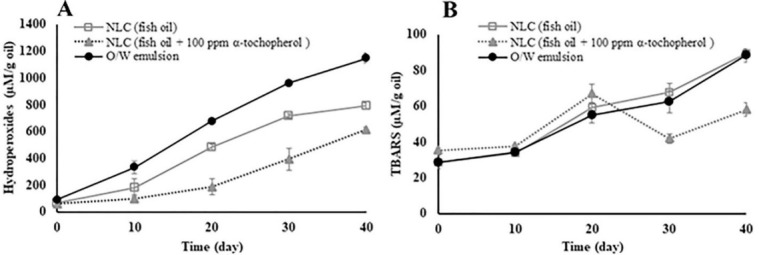
Formation of (A) lipid hydroperoxides and (B) Thiobarbituric acid reactive substances (TBARS) in lipid particles during storage at 25°C. Data points and error bars represent means (n = 3) ± standard deviations.

The changes in primary oxidation products, i.e. lipid hydroperoxides, and in secondary oxidation products, i.e. TBA, of lipid NPs were measured at 25°C over 40 days (Figure 5). It should be noted that the heating of omega-3 fish oil during the preparation of emulsions did not lead to a fast increase in lipid oxidation reactions. This suggests that the heating time was too short to initiate degradation of the un-oxidized omega-3 fish oil (low PV was found in the bulk material prior to preparation). Previous studies showed that the degradation of PUFAs is insignificant in the temperature below than 80–100°C when applied for a short time.^17^

As shown in Figure 5a, the results of the oxidative stability tests showed a difference in PV between emulsion and NLCs. The PVs of the NLC formulations was less than fish oil-in-water emulsion formulation during storage at 25°C. The observed difference at various storage days was significant (*P *≤ 0.05) except the first day. The PVs of the NLC formulations was almost lower than those of the fish oil-in-water emulsion demonstrating the NLC is an effective protection system. Although no significant difference (*P* ≥ 0.05) was observed in the secondary products between samples of a fish oil-in-water emulsion and the NLCs in the early days, there was a significant difference (*P* ≤ 0.05) in the final days (Figure 5b).

The encapsulation of omega-3 fish oil in the NLC showed more oxidative stability than the formulation of omega-3 fish oil in the emulsion. These results are in a good agreement with the previous studies.^15,17^ In NLCs containing fish oil, a Precirol ATO5 shell is most likely formed around the omega-3 fish oil through heterogeneous crystallization in the solidified interfacial layer. This shell may possibly act as a physical barrier which inhibits oxidation by limiting the access of oxygen, pro-oxidants, and light. In o/w emulsion (containing 100% fish oil), this protective shell is missing, so that the oxygen, pro-oxidants, and light can interact to a greater degree.^12,17^ Salminen et al^17^ achieved the same results with the present work by encapsulation of fish oil in the NLC. Also, Krasodomska et al^15^ used PUFA-rich oils of blackcurrant, blackberry, raspberry, strawberry, and plum as components of NLCs. Their results showed that the NLC was an effective method for increasing oxidative stability of the PUFAs.

According to Figure 5, the results of the oxidative stability tests (PV and TBARS) showed that the co-encapsulation of fish oil and α-tocopherol in the NLC could increase oxidative stability of lipid particles. Chen et al^20^ showed that with co-encapsulation of three hydrophobic bioactive compounds including fish oil, phytosterols, and limonene, the preservation of EPA and DHA in the co-encapsulated omega-3 fish oil was higher than the encapsulated fish oil. Also, they found that the co-encapsulated omega-3 fish oil had better flavor than the encapsulated omega-3 fish oil after drying and storage.

In addition, our findings showed that α-tocopherol especially could significantly inhibit the formation of secondary products (TBARS) in the final days (*P* ≤ 0.05). This result suggests that α-tocopherol could prevent the formation of secondary oxidation products. Salminen et al^17^ reported that by encapsulation of fish oil in the NLC, the formation of lipid hydroperoxide (primary product), propanol and hexanal (secondary product) compared to fish oil in water emulsion, 72%, 53%, and 57%, was decreased, respectively. These results suggest that encapsulation of omega-3 fish oil in NLC was probably more effective to decrease of the primary oxidative products than the secondary products. In addition, according to the omega-3 fish oil release results, there was some non-encapsulated omega-3 fish oil in the matrix. Therefore, some oxidation products are related to free fish oil, and the encapsulation method has no effect on decreasing the oxidation rate of non-encapsulated fish oil.

Based on these results, it can be stated that the encapsulation methods such as NLC can further prevent the formation of primary products to form secondary products. It is believed that for starting oxidation and production of the primary oxidation products, oxygen is needed.^30^ This result can be attributed to the effect of preventing oxygen to the oil surface of NPs due to protective shell obtained from encapsulation. On the other hand, the approach of adding an antioxidant such as α-tocopherol can prevent to increase in both primary and secondary products due to the presence in the oil matrix. Based on these findings, it can be stated that the co-encapsulation omega-3 fish oil with an antioxidant in NLC is a suitable approach to obtain a good oxidative stability for omega-3 fish oil.

## Conclusion

In this study, we formulated a stable omega-3 fish oil-loaded NLC using Precirol ATO5 as a solid lipid phase and poloxamer 407 as an emulsifier. The formulated NPs were stable in RT, and also their thermal behavior indicated that the ingredients including an emulsifier, solid phase, and liquid phase were mixed very well and created a new structure. The results of oxidative stability showed that oxidation changes in the NLCs were lower than the o/w emulsion. In addition, co-encapsulation of omega-3 fish oil/α-tocopherol in NLC showed more oxidative stability than the NLC containing only fish oil. Our results indicated that encapsulation of omega-3 fish oil in the NLC was more effective to decrease of the primary oxidative products than the secondary products. Our findings showed that co-encapsulation of fish oil and α-tocopherol within the NLC can be an appreciate strategy in order to fortify the foodstuffs especially the transparent beverages by omega-3 fish oil. However, further studies are necessary to investigate the effects of other factors on the oxidative stability of omega-3 fish oil in the formulation of beverages.

## Ethical Issues

Not applicable.

## Conflict of Interest

Authors declare that there is no conflict of interest regarding the publication of this article.

## Acknowledgments

The authors would like to thank the Shahrood University of Technology through the financial support of this work.

## References

[1] Mark-Herbert C (2004). Innovation of a new product category—functional foods. Technovation.

[2] Yazicioglu B, Sahin S, Sumnu G (2015). Microencapsulation of wheat germ oil. J Food Sci Technol.

[3] Piorkowski DT, McClements DJ (2014). Beverage emulsions: Recent developments in formulation, production, and applications. Food Hydrocoll.

[4] Mozaffarian D, Wu JH (2011). Omega-3 fatty acids and cardiovascular disease: effects on risk factors, molecular pathways, and clinical events. J Am Coll Cardiol.

[5] Zhang Z, Decker EA, McClements DJ (2014). Encapsulation, protection, and release of polyunsaturated lipids using biopolymer-based hydrogel particles. Food Res Int.

[6] Pham-Huy LA, He H, Pham-Huy C (2008). Free radicals, antioxidants in disease and health. Int J Biomed Sci.

[7] Halwani M, Yebio B, Suntres ZE, Alipour M, Azghani AO, Omri A (2008). Co-encapsulation of gallium with gentamicin in liposomes enhances antimicrobial activity of gentamicin against Pseudomonas aeruginosa. J Antimicrob Chemother.

[8] Mirhosseini H, Tan CP, Taherian AR (2008). Effect of glycerol and vegetable oil on physicochemical properties of Arabic gum-based beverage emulsion. Eur Food Res Technol.

[9] Fathi M, Mozafari MR, Mohebbi M (2012). Nanoencapsulation of food ingredients using lipid based delivery systems. Trends Food Sci Technol.

[10] Muller RH, Radtke M, Wissing SA (2002). Nanostructured lipid matrices for improved microencapsulation of drugs. Int J Pharm.

[11] Sagalowicz L, Leser ME (2010). Delivery systems for liquid food products. Curr Opin Colloid Interface Sci.

[12] Tamjidi F, Shahedi M, Varshosaz J, Nasirpour A (2013). Nanostructured lipid carriers (NLC): A potential delivery system for bioactive food molecules. Innov Food Sci Emerg Technol.

[13] Pardeike J, Hommoss A, Muller RH (2009). Lipid nanoparticles (SLN, NLC) in cosmetic and pharmaceutical dermal products. Int J Pharm.

[14] Joshi MD, Muller RH (2009). Lipid nanoparticles for parenteral delivery of actives. Eur J Pharm Biopharm.

[15] Krasodomska O, Paolicelli P, Cesa S, Casadei MA, Jungnickel C (2016). Protection and viability of fruit seeds oils by nanostructured lipid carrier (NLC) nanosuspensions. J Colloid Interface Sci.

[16] Salminen H, Helgason T, Kristinsson B, Kristbergsson K, Weiss J (2013). Formation of solid shell nanoparticles with liquid omega-3 fatty acid core. Food Chem.

[17] Salminen H, Aulbach S, Leuenberger BH, Tedeschi C, Weiss J (2014). Influence of surfactant composition on physical and oxidative stability of Quillaja saponin-stabilized lipid particles with encapsulated ω-3 fish oil. Colloids Surf B Biointerfaces.

[18] Chaiyasit W, McClements DJ, Weiss J, Decker EA (2008). Impact of surface-active compounds on physicochemical and oxidative properties of edible oil. J Agric Food Chem.

[19] Tikekar RV, Nitin N (2011). Effect of physical state (solid vs liquid) of lipid core on the rate of transport of oxygen and free radicals in solid lipid nanoparticles and emulsion. Soft Matter.

[20] Chen Q, McGillivray D, Wen J, Zhong F, Quek SY (2013). Co-encapsulation of fish oil with phytosterol esters and limonene by milk proteins. J Food Eng.

[21] Neupane YR, Srivastava M, Ahmad N, Kumar N, Bhatnagar A, Kohli K (2014). Lipid based nanocarrier system for the potential oral delivery of decitabine: formulation design, characterization, ex vivo, and in vivo assessment. Int J Pharm.

[22] Wang LJ, Hu YQ, Yin SW, Yang XQ, Lai FR, Wang SQ (2015). Fabrication and characterization of antioxidant pickering emulsions stabilized by zein/chitosan complex particles (ZCPs). J Agric Food Chem.

[23] Liu CH, Wu CT (2010). Optimization of nanostructured lipid carriers for lutein delivery. Colloids Surf A Physicochem Eng Asp.

[24] Trotta M, Debernardi F, Caputo O (2003). Preparation of solid lipid nanoparticles by a solvent emulsification-diffusion technique. Int J Pharm.

[25] Zirak MB, Pezeshki A (2015). Effect of surfactant concentration on the particle size, stability and potential zeta of beta carotene nano lipid carrier. Int J Curr Microbiol Appl Sci.

[26] Lason E, Sikora E, Ogonowski J (2013). Influence of process parameters on properties of Nanostructured Lipid Carriers (NLC) formulation. Acta Biochim Pol.

[27] Kovacevic AB, Muller RH, Savic SD, Vuleta GM, Keck CM (2014). Solid lipid nanoparticles (SLN) stabilized with polyhydroxy surfactants: Preparation, characterization and physical stability investigation. Colloids Surf A Physicochem Eng Asp.

[28] Ghosh Chaudhuri R, Paria S (2012). Core/shell nanoparticles: classes, properties, synthesis mechanisms, characterization, and applications. Chem Rev.

[29] Carbone C, Campisi A, Manno D, Serra A, Spatuzza M, Musumeci T (2014). The critical role of didodecyldimethylammonium bromide on physico-chemical, technological and biological properties of NLC. Colloids Surf B Biointerfaces.

[30] Akoh CC, Min DB. Food lipids: chemistry, nutrition, and biotechnology. New York: CRC Press; 2008.

